# Reverse Zoonosis of COVID-19: Lessons From the 2009 Influenza Pandemic

**DOI:** 10.1177/0300985820979843

**Published:** 2020-12-09

**Authors:** Syriam Sooksawasdi Na Ayudhya, Thijs Kuiken

**Affiliations:** 1 Erasmus University Medical Centre, Rotterdam, Netherlands

**Keywords:** reverse zoonoses, pandemic, pandemic H1N1 influenza, coronavirus disease 2019, host species barriers, review, species jumps

## Abstract

Over the past decade, pandemics caused by pandemic H1N1 (pH1N1) influenza virus in 2009 and severe acute respiratory syndrome virus type 2 (SARS-CoV-2) in 2019 have emerged. Both are high-impact respiratory pathogens originating from animals. Their wide distribution in the human population subsequently results in an increased risk of human-to-animal transmission: reverse zoonosis. Although there have only been rare reports of reverse zoonosis events associated with the ongoing coronavirus disease 2019 (COVID-19) pandemic from SARS-CoV-2 so far, comparison with the pH1N1 influenza pandemic can provide a better understanding of the possible consequences of such events for public and animal health. The results of our review suggest that similar factors contribute to successful crossing of the host species barriers in both pandemics. Specific risk factors include sufficient interaction between infected humans and recipient animals, suitability of the animal host factors for productive virus infection, and suitability of the animal host population for viral persistence. Of particular concern is virus spread to susceptible animal species, in which group housing and contact network structure could potentially result in an alternative virus reservoir, from which reintroduction into humans can take place. Virus exposure in high-density populations could allow sustained transmission in susceptible animal species. Identification of the risk factors and serological surveillance in SARS-CoV-2-susceptible animal species that are group-housed should help reduce the threat from reverse zoonosis of COVID-19.

Emerging viral diseases are an important threat for public health. Manyof these diseases are zoonotic, in the sense that their original source is animals. Sporadically, these emerging viral diseases can lead to pandemics in humans. When such a pandemic occurs, the high number of infected people can in turn form a source of infection for animals: reverse zoonosis. There are 2 main concerns about such reverse zoonosis events. First, the infected animals can become ill and even die; second, the population of animals in question can become a virus reservoir, from which reintroduction into humans can take place.

For any virus spillover between species, including reverse zoonosis, several barriers need to be breached.^24^ There must be sufficient contact between donor species (in this case, an infected human being) and recipient species and enough compatibility between the virus and the new host to allow replication and the possibility of transmission to other members of the recipient species. If this transmission can occur, the contact network structure of the recipient species, together with variations in transmission through this network, are critical in determining whether the virus will persist or die out.

Currently, the pandemic of coronavirus disease 2019 (COVID-19) is ongoing, and the causative virus, severe acute respiratory syndrome virus type 2 (SARS-CoV-2), is becoming more widely distributed in the human population.^78^ Already there are several reports of reverse zoonosis events, involving domestic cats and dogs, tigers, lions, and American mink (Fig. 1).^37,38,42,45,60^ To get a better understanding about the possible consequences of such events for public health and animal health, we think that it is worthwhile to review what happened during the H1N1 influenza pandemic (pH1N1) in 2009 (Fig. 2). Although pH1N1 influenza is caused by a different virus, it is similar to COVID-19 in that it targets the respiratory tract and is often transmitted by respiratory droplets. Importantly, there is an overlap in host range of the 2 viruses. We also present clinical features, necropsy, and histopathological findings of SARS-CoV-2 and pH1N1 virus infection in different animal species in order to raise awareness of people in the field on the inclusion of these virus infections in their differential diagnosis during the ongoing pandemic period.

**Figure 1.**
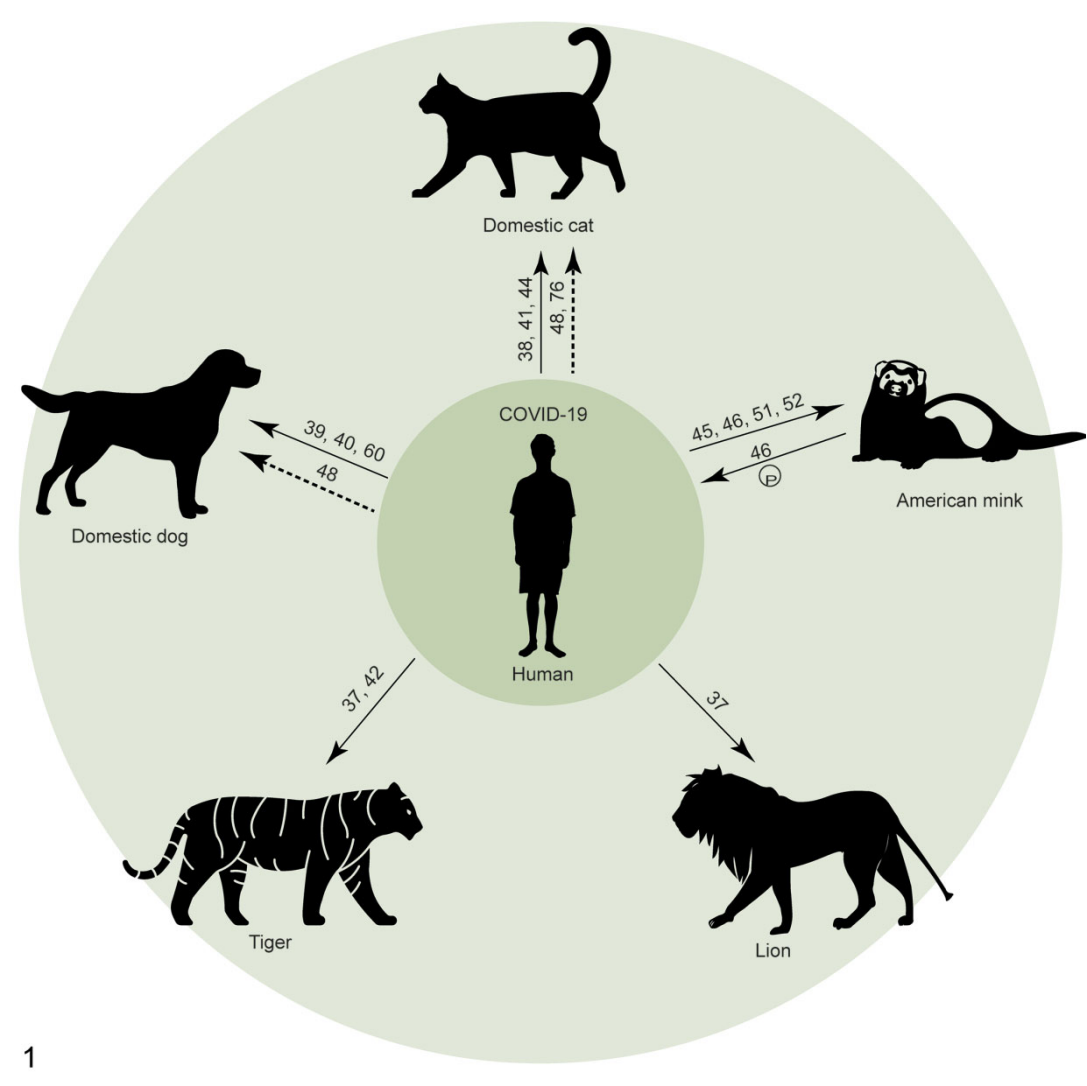
Reverse zoonosis events of coronavirus disease 2019 (COVID-19). Numbers indicate the reference of the publication or report. Arrows pointing from human to animal represent reverse zoonosis events. Solid arrows represent likely human-to-animal transmission confirmed by viral RNA, sequencing data, or virus isolation. Dashed arrows represent possible human-to-animal transmission showed by serological data. “P” represents persistent infection in an animal host species.

**Figure 2.**
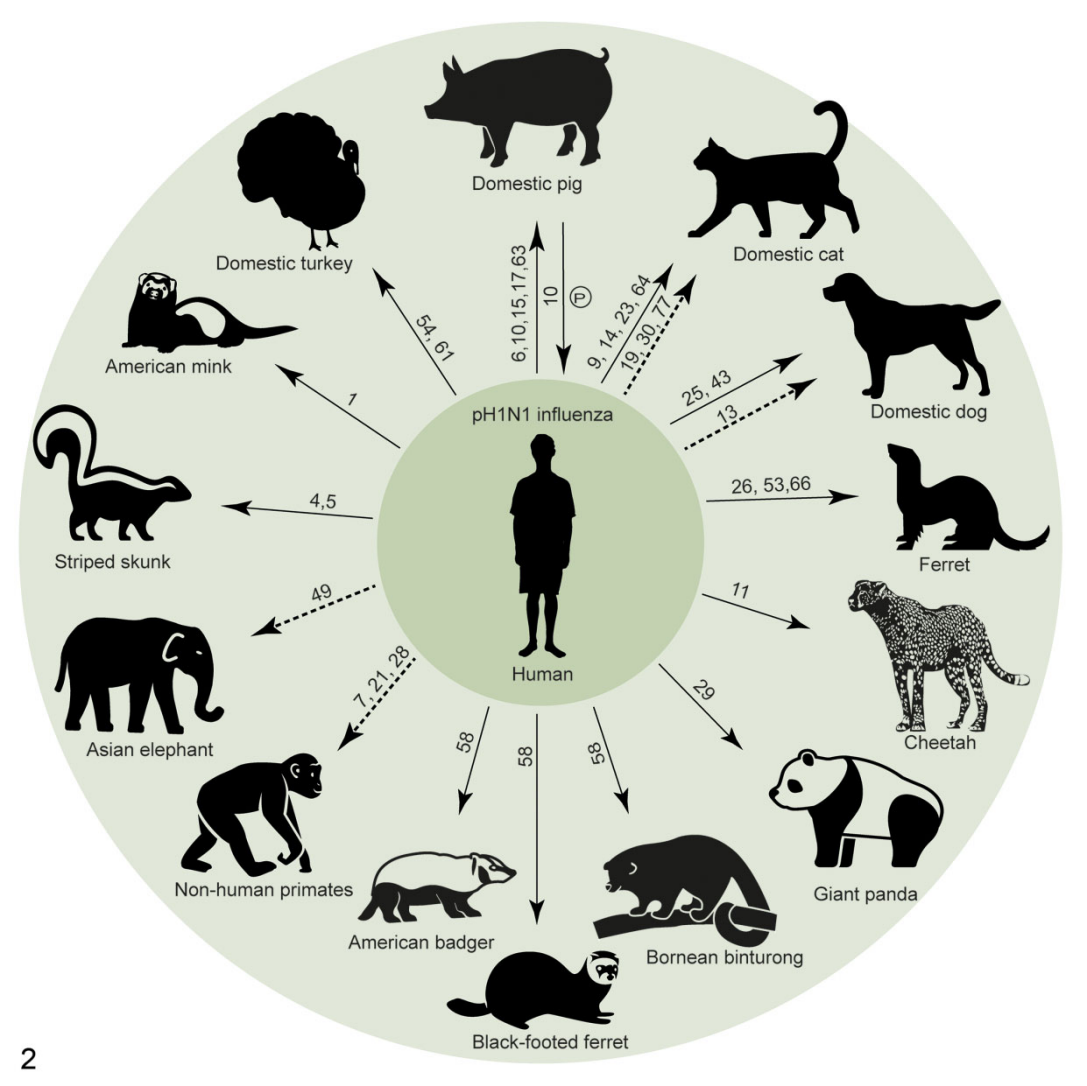
Reverse zoonosis events of pandemic H1N1 (pH1N1) influenza. Numbers indicate the reference of the publication or report. Arrows pointing from human to animal represent reverse zoonosis events. Arrows pointing from animal to human represent “reverse-reverse zoonosis” events. Solid arrows represent likely human-to-animal transmission confirmed by viral RNA, sequencing data, or virus isolation. Dashed arrows represent possible human-to-animal transmission showed by serological data. “P” represents persistent infection in an animal host species.

## Pandemic H1N1 Influenza

### Farm Animals

Events of human-to-pig transmission were the most frequently reported reverse zoonosis of pH1N1 influenza. The first cases of human-origin pH1N1 virus infections in a pig farm were reported in Canada just 1 month after pH1N1 influenza had spread worldwide in the human population.^18^ Epidemiologic evidence for human-to-pig transmission was based on pigs testing positive by qPCR (quantitative polymerase chain reaction) for pH1N1 virus RNA after contact with farmers who were infected with pH1N1 virus.^15,17^ Sequencing analysis showed that the viruses infecting humans and pigs were highly similar, indicating no virus adaptation was necessary for replication in pigs.^15,63^ Since then, pH1N1 virus has repeatedly spread from human to pigs globally, resulting in genetic diversity of pH1N1 viruses in pig populations.^35^ In pigs infected with pH1N1 virus, mild or asymptomatic respiratory diseases were observed. Histopathologic changes consisted of multifocal broncho-interstitial pneumonia with suppurative alveolitis, suggesting secondary bacterial coinfections. Sustained transmission between pigs has been reported in both natural and experimental studies,^6,50,67^ suggesting that pigs can act as a reservoir for pH1N1 virus.

The domestic turkey is another food animal species in which reverse zoonosis of pH1N1 influenza has occurred. Sporadic infections of pH1N1 virus have been reported mainly in turkey breeder flocks. Human-to-turkey transmission of pH1N1 virus potentially occurred during artificial insemination (AI), based on the evidence that farm workers had been sick with flu-like symptoms during the AI period and subsequently had antibodies against pH1N1 virus. Phylogenetic analysis of pH1N1 virus isolated from infected farm workers and turkeys were grouped in the same cluster, supporting human-to-turkey transmission.^61^ Evidence of pH1N1 antibody in serum, viral RNA from oropharyngeal and cloacal swab specimens and virus isolation from specific pathogen free fowl embryonated eggs indicated pH1N1 influenza in turkey breeder flocks.^54,61^ No to mild clinical signs except for reduced numbers of eggs were observed in affected flocks.^47,54,61^ pH1N1 virus was detected in reproductive tissues as well as the cecal tonsils and bursa of Fabricius, and no lesions were detected in other tissues.^47,61^ This corresponds to experimental infections, where turkeys developed clinical signs only after intracloacal but not after intranasal inoculation with pH1N1 virus, even though intranasal inoculation is a successful route of pH1N1 virus inoculation in other species.^47^ Remarkably, besides domestic turkeys, pH1N1 virus infections have not been reported in other avian species.

pH1N1 virus infections in American mink (*Neovison vison*) were first reported in a mink farm in Norway during the pandemic. Although the source of transmission was unclear, phylogenetic analysis of sequences derived from affected mink were highly similar to human derived-isolates during the pH1N1 pandemic. The infection might have been transmitted from subclinically infected farm workers or virus-contaminated feed from infected pig offal. The infected mink kits developed severe respiratory disease and had increased mortality rates. Pathological changes included acute to subacute interstitial pneumonia with edema, lymphocyte infiltration, and epithelial hyperplasia. pH1N1 virus antigen was mainly observed in the nuclei of the epithelial lining of bronchi and bronchioles and also of pneumocytes in the alveolar septa.^1^

### Pets

Many cases of pH1N1 virus infection were documented in domestic dogs, domestic cats, and pet ferrets. pH1N1 viral RNA was detected from nasal swab and pharyngeal specimens, and from necropsy tissues including tonsils, trachea, and lungs.^9,25^ Phylogenetic analysis of isolated pH1N1 virus from affected animals revealed a close relationship to pH1N1 virus in humans during the pandemic.^14,26,66^ Influenza-like illness was reported from family members prior to signs of influenza-like illness in affected animals.^9,26,43,66^ In addition, influenza A virus was detected by influenza A virus rapid test in a family member before animals exhibited respiratory signs.^66^ Thus, the chronology of events and the detection of influenza A virus in a family member suggest that infected owners were the source of infection in dogs, cats, and ferrets in their household. Due to the close contact between owners and their companion animals, serological surveillance was performed. Antibodies against pH1N1 virus were detected in cats and dogs during the period of virus spread in the human population. Although the exact mode of transmission is unclear, it is possible that this occurred due to human-to-animal transmission, since pet animals frequently live together and are in close contact with humans.^13,77^ Serological studies showed that group housing of animals likely facilitated efficient intraspecies transmission, including cat-to-cat transmission and ferret-to-ferret transmission.^3,8,14^ However, pH1N1 virus transmission between dogs seemed to be limited.^25^ Although all these species were susceptible for pH1N1 virus infection, clinical signs varied among them. While cats and ferrets often developed severe respiratory signs, including dyspnea, coughing, and sneezing, and even died from the infection,^8,9,23,43,53,64^ dogs either showed no clinical signs or only mild respiratory signs, such as rhinorrhea and coughing.^25,43^ Correlated to the severity of disease, pathological changes in fatally infected cats and ferrets consisted of multifocal severe necrotizing broncho-interstitial pneumonia,^27,62^ while no obvious lesions were observed in dogs.^25^

### Captive Wild Animals

pH1N1 virus was reported to infect wild animals maintained in captivity, nearly all of which were carnivores held in zoos. Several zoo carnivores including a cheetah (*Acinonyx jubatus*), a Bornean binturong (*Arctictis binturong penicillatus*), an American badger (*Taxidea taxus*), a black-footed ferret (*Mustela nigripes*), and a giant panda (*Ailuropoda melanoleuca*) were susceptible to pH1N1 virus infection.^11,29,58^ The source of infection in these cases was not determined due to lack of availability of clinical samples from humans with close contact. However, all affected animals were in contact with caretakers or veterinarians, and were housed separately from other wildlife.^11,29,58^ This suggests that animals may have been infected by humans even though the latter did not show clinical symptoms. Although no viral RNA detection or virus isolation from the potential human sources of virus were performed, phylogenetic analysis showed that viruses isolated from infected animals were highly similar to pH1N1 virus circulating among humans during the pandemic and the subsequent seasonal influenza period.^11^ While the cheetah, the Bornean binturong, the American badger, and the giant panda exhibited severe respiratory signs of infection—hematopnea and dyspnea—no clinical signs were reported in the black-footed ferret.^11,29,58^

Besides in zoo carnivores, there was evidence of pH1N1 virus infection in Asian elephants (*Elephas maximus*) based on the presence of pH1N1 antibodies. The source of infection remains unknown, but most likely was infected mahouts, or infected tourists who attended activities such as elephant riding and feeding.^49^ Similarly to Asian elephants, pH1N1 virus–specific antibodies in nonhuman primates have been reported in several studies. However, there is no evidence that nonhuman primates had clinical signs of disease or mortality from pH1N1 virus infection.^7,21,28^ Whether animal-to-animal transmission can be sustained among captive wild animals, with the risk of becoming a new reservoir, remains unknown since serological and epidemiological studies in captive wild animals are limited.

### Free-Living Wild Animals

The only free-living wild animal species in which pH1N1 virus has been reported is the striped skunk (*Mephitis mephitis*). Sequencing and phylogenetic analysis of virus isolated from affected animals were highly related to pH1N1 virus circulating in humans.^4,5^ The source of infection was unclear. In one study, the skunks lived near a mink farm, suggesting that spillover of pH1N1 virus from infected mink farm workers or infected mink may have occurred.^4^ In the other study, the skunks were found in an urban park where hand feeding by park visitors normally took place.^5^ Clinical signs were not noted, but purulent nasal exudate was observed in fatally infected skunks. Histopathological changes ranged from moderate, acute, suppurative rhinitis to severe bronchopneumonia.^4,5^

## Coronavirus Disease 2019

A decade after pandemic H1N1 influenza, the newly emerged COVID-19 caused by SARS-CoV-2 infection has been reported in a number of farm, pet, and wild animal species, both in natural circumstances and experimental settings. In some cases, human-to-animal transmission of this virus has impacted animal welfare and caused financial loss.

### Farm Animals

American mink have tested positive for SARS-CoV-2 viral RNA in several mink farms in European countries including the Netherlands, Denmark, and Spain.^45,51,52^ A study of the outbreak in mink farms in the Netherlands reported that some farm workers had respiratory symptoms prior to SARS-CoV-2 outbreaks in the farms. Viral RNA was detected in throat and rectal swabs from affected mink by qPCR. In addition, viral RNA was detected in dust particles suggesting indirect transmission between mink via fomites or droplets produced by affected mink. Importantly, serological surveillance was performed in which 60 random serum samples were collected from the outbreak mink farms. All mink, except one sample from one mink farm, had seroconverted against SARS-CoV-2 as tested by neutralization assay indicating previous infections were widespread in the mink populations.^45^ This indicates that the virus was originally transmitted from humans to mink, and that there was subsequent sustained transmission among the mink. Additionally, employees, who had tested negative for SARS-CoV-2 RNA 2 weeks previously, developed respiratory symptoms and tested positive for SARS-CoV-2 viral RNA at the same time that mink were diagnosed. Subsequently, whole-genome sequencing and phylogenetic analysis showed that the sequences from affected employees were in the same cluster as sequences derived from the mink.^46^ Together, data from timing of infection, whole-genome sequencing and phylogenetic analysis indicate that the virus was transmitted from mink to humans, as a so-called “reverse-reverse zoonosis.” SARS-CoV-2 antibodies were detected in domestic cats living on the mink farms, indicating that they had been infected with the virus.^45^ Thus, it could be that domestic cats played a role in the spread of the virus. The infected mink mostly developed watery nasal discharge, and some developed severe respiratory illness. Pathologic changes in dead mink were severe acute interstitial pneumonia.^31,45^ Besides in American mink, reverse zoonosis of COVID-19 has not been documented in other farm animal species so far.

### Pets

Domestic dogs and cats from households with either confirmed human cases of COVID-19 or asymptomatic SARS-CoV-2 infection have been reported to be infected with SARS-CoV-2, indicating the potential of virus transmission from humans to these species. Two out of 17 domestic dogs from owners with SARS-CoV-2 infection were infected with SARS-CoV-2, and did not show respiratory signs. A low load of viral RNA was detected in nasal swabs, but not in fecal swabs from these dogs. Sequences of viruses from 2 dogs showed strong similarity to the virus isolated from the human cases, suggesting human-to-animal transmission. Both infected dogs seroconverted, based on plaque reduction neutralization assays.^60^ In addition, a domestic dog in the United States was reported to have antibodies against SARS-CoV-2, suggesting exposure; no viral RNA was detected in samples from this dog.^39^ Serum from another dog in the same household tested negative for SARS-CoV2 neutralizing antibodies, suggesting dog-to-dog transmission is limited.^40^

In domestic cats, several reports showed SARS-CoV-2 infections in cats belonging to a SARS-CoV-2-infected owner or a SARS-CoV-2-infected neighbor. Viral RNA was detected from respiratory samples and gastrointestinal samples—including vomitus and stool—of the cats, but infectious virus could not be isolated. Sequencing analysis confirmed SARS-CoV-2 infection. Affected cats developed a wide range of clinical signs, from mild to severe respiratory signs, as well as gastrointestinal signs. Sneezing and ocular discharge were observed in mild respiratory illness^38^ while dyspnea was found in severe respiratory cases.^41^ Vomiting was observed in an infected cat with gastrointestinal signs.^44^ Most of infected animals fully recovered, suggesting mild disease. However, some animals died, likely due to other underlying diseases.^41^

### Captive Wild Animals

SARS-CoV-2 infections in captive wild animals were reported from 2 enclosures at a zoo. Viral RNA was first detected in a nasal swab of a tiger (*Panthera tigris*) with respiratory signs such as dry cough and wheezing.^42^ Subsequently, another 3 tigers and 2 lions (*Panthera leo*) at the same facility were all confirmed with SARS-CoV-2 infection, based on viral RNA detection in fecal samples.^37^ Sequencing analysis showed that viruses from infected animals were identical to SARS-CoV-2 in humans. These data suggest that the virus could have been transmitted by a zookeeper who might not have developed symptoms of COVID-19 at the time of exposure to these animals.^74^ Whether subsequent animal-to-animal transmission occurred is not known.

## Experimental Studies

Experimental studies have shown that a number of animal species support SARS-CoV-2 infection. In domestic cats, ferrets, rhesus macaques, and cynomologus macaques, high viral RNA levels were detected by qPCR, indicating that the virus infected and replicated efficiently in the respiratory tract of animals without causing severe disease or death.^22,33,56,59^ However, upon experimental infection, golden hamsters exhibited severe clinical signs and pathological changes of severe interstitial pneumonia.^20^ Evidence of animal-to-animal transmission has been shown by detection of viral RNA in sentinel animals after having direct or indirect contact with virus-inoculated animals. In these transmission studies, sentinel cats and ferrets were infected by SARS-CoV-2 via airborne and direct-contact transmission.^16,22,55,59^ In contrast, low viral RNA levels were detected in swabs collected from dogs inoculated according to the same methods, suggesting they were less susceptible to SARS-CoV-2 infection. In domestic pigs, chickens and domestic ducks, no viral RNA was detected from any swabs and the animals remained seronegative for 2 weeks post inoculation. These data suggest that these livestock species were not susceptible to SARS-CoV-2 infection.^59^

## Host Species Barriers

Interaction of several factors are involved in limiting transmission of a virus infection from a donor to a recipient host species; these represent the host species barriers to virus infections. Thus, generally, viruses only sporadically jump from one species to another. Specific interactions are required to accomplish such species jumps and sustain transmission.^24^ In the first place, sufficient interaction between donor host and recipient host is important, and this is a common factor for successful species jumps in both pH1N1 and SARS-CoV-2 transmissions. Seroprevalence of pH1N1 in cats in different cities in northeastern China revealed a higher prevalence rate of pH1N1 in pet cats (30.6%) compared to roaming cats (11%) tested by neutralization assay.^77^ Similar to pH1N1, neutralization titer in pet cats owned by COVID-19 patients was higher than the titer from pet hospital cats and stray cats.^76^ These observations from serological surveillances suggest a likely transmission from infected owners to their pets by close contact, thus addressing the role of sufficient interaction between donor hosts and recipient hosts in crossing the species barrier. Although 2 host species share the same geographical area and habitat, host behavior can either limit or enhance pathogen transmission. Certain behaviors of humans, which enhance close contact between infected owners or keepers and their animals, increase the chance of reverse zoonosis. For example, artificial insemination of domestic turkeys likely caused pH1N1 virus spillover from infected humans to domestic turkeys. It could be that particular procedures of caretakers in mink farms, such as weaning pups and vaccination, may have led to human-to-mink transmission. Also, hand-feeding by visitors, surface contamination of bedding or other fomites, or contaminated food could be sources of spillover from humans to animals.^5,45^ After the global spread of pH1N1 virus in 2009, the virus continued to circulate in humans, resulting in normal seasonal epidemics of influenza. This contributed to repeated virus introductions from humans to susceptible animals. Multiple events of human-to-pig transmission occurred worldwide during 2009 to 2014.^18,36^ The introduction of human pH1N1 viruses into the pig populations and subsequent co-circulation with endemic swine influenza viruses resulted in an increase of genetic diversity by exchange of genome segments.^35^ For example, a novel reassortment (A/swine/Hong Kong/201/2010 [H1N1]) was found during virological surveillance. This reassortant was composed of a neuraminidase (NA) gene from pH1N1, a hemagglutinin (HA) gene from a European avian-like H1 virus, and the 6 internal genes from triple reassortant H1N1 viruses.^72^ If the new reassortments result in increased transmission, virulence, or immune escape, they may cause a massive threat to humans and public health by potential generation of a new pandemic influenza virus.^57,75^

Not much is known about the association between genetic diversity and pathogenicity during SARS-CoV-2 outbreaks in animals. Whole-genome sequencing analysis in SARS-CoV-2-infected mink revealed high genetic diversity in farms which tested negative before, suggesting a fast evolution of viruses in the mink populations. To date, no specific mutations have been observed that are common to all mink isolates. Even though one specific substitution (D614G) associated with increased virulence in vitro was present in some farm clusters, no clear differences in clinical signs, virulence, or transmissibility to humans were found.^46^ Further surveillance and sequencing analysis are required in order to monitor amino acid substitutions that may be associated with changes in disease severity or transmission. In some cases, both pH1N1 virus and SARS-CoV-2 infections caused relatively no or only mild signs, and virus was transmitted before clinical symptoms became apparent.^15,45^ These could facilitate under-detection of human-to-animal transmission.

In the second place, virus-host interaction is important in determining susceptibility of a new host species to a virus, and virus transmission to other individuals in the new host species. Similarity of biological host factors between humans and animals (for instance, receptor expression, proteases, and enzymes) can partially determine the potential of a virus to switch species. For example, pH1N1 virus preferentially attaches to α2,6-linked sialic acids, which are abundantly expressed in the upper respiratory tract of animals including pigs, cats, and ferrets.^12,34,73^ Angiotensin converting enzyme 2 (ACE2), a receptor for SARS-CoV-2, is expressed in tissues of cats and ferrets.^70^ Once the virus attaches to a new host cell, compatibility between virus proteins and host cell machinery is required for efficient virus replication and potential transmission. Viruses that replicate to a high level are generally more easily transmitted to other hosts. For example, pH1N1 virus replicates efficiently in pigs, cats, and ferrets, allowing subsequent transmission.^32,71^ Similarly to pH1N1 virus infections, SARS-CoV-2 replicates efficiently in cats and ferrets resulting in transmission to sentinel animals. In contrast, low replication efficiency limits virus transmissibility. For example, limited SARS-CoV-2 replication in dogs and absence of virus replication in pigs and chickens are associated with limited or no transmission.^59^

In the third place, if the transmission can occur, intraspecific contacts in the recipient population are crucial in determining whether the virus will persist or die. The possibility of maintenance of infection in a new host species depends in a complex way on the population sizes and the degrees of mixing of donor and recipient host species as well as the rate of virus transmission in each.^24^ Several lines of evidence in both pH1N1 virus and SARS-CoV-2 infections suggest that group-housed animals have a higher chance of spreading the viruses to other individuals of the same species, compared to animals kept individually. High population density, high farm density, and large herd size are the most common risk factors for influenza virus infection in pig farms.^2,65,68^ The same risk factors could also be valid for efficient SARS-CoV-2 virus transmission in mink farms. In human COVID-19 patients, generation interval or time between infection events in a donor and recipient pair is around 4 to 5 days; however, high dose of virus exposure in a high-density population could potentially reduce this interval resulting in broad spread of infections in mink farms.^46^ Several examples of efficient animal-to-animal transmissions have been reported in a cat colony, a mink farm, and a pig farm.^14,15,45^

## Epidemiology and Serological Surveillance in Reverse Zoonosis Events

For the assessment of risk for animals and humans involved with reverse zoonosis outbreaks of pH1N1 or COVID-19, a combination of clinical, epidemiological, sequencing analysis, and laboratory investigations are needed. Particularly, some infected animals show no or only mild clinical signs, which can make it difficult to detect or apply quarantine measures. However, those animals can develop antibodies against these virus infections. Thus, conducting seroepidemiological studies in outbreak areas can assist in identifying susceptible animals and transmission within the population.^15^ These approaches are also used to investigate reverse zoonosis in large-scale outbreaks, for example, pH1N1 transmission between pig farms and clustering of SARS-CoV-2 outbreaks in 16 mink farms within the same province.^45,46,69^ Together with chronology of infection, serologic evidence for infection with pH1N1 or COVID-19 in employees and workers in outbreak areas point out the occupational risk of human-to-animal transmission and vice versa.^10,46^

Once the reverse zoonosis events occur, it is crucial to know whether infection is maintained in an animal population and has a potential to spillover back to humans. Therefore, continued serosurveillance in susceptible animals and other animal species in the same area are recommended. For example, increased seroprevalence of pH1N1 virus in cats after the 2009 pandemic was detected by serosurveillance studies suggested sustained transmission of this virus in cat populations.^19,30,77^ To date, few serosurveillance studies have been performed for SARS-CoV-2 in domestic dogs, cats, and mink, indicating a need for further serosurveillance studies in human-animal interfaces that represent a critical point for cross-species transmission.^45,46,48,76^

## Conclusions

Although the number of reported reverse zoonosis events of COVID-19 are limited so far, sufficient contact between humans and animals, compatibility between SARS-CoV-2 and the new animal host, group housing of large numbers of animals, and the structure of the animal host’s contact network can overcome the host species barriers. In order to assess the risk of reverse zoonosis of COVID-19, it is crucial to determine whether factors that allow the host species barriers to be overcome are present for those situations where SARS-CoV-2-infected humans are in contact with animals. Additionally, knowledge of clinical and pathological features of SARS-CoV-2 infection in different animal species will raise awareness of the possibility of this diagnosis during the ongoing pandemic period. Following the pandemic, serological surveillance in animal populations at risk should be conducted, particularly in group-housed animals. Together, this knowledge will improve our understanding of the potential risk for reverse zoonosis of COVID-19.
